# Decoding microglial aging through multi-model approaches

**DOI:** 10.4103/NRR.NRR-D-25-00229

**Published:** 2025-06-19

**Authors:** Martin Škandík, Bertrand Joseph

**Affiliations:** Toxicology Unit, Institute of Environmental Medicine, Karolinska Institutet, Stockholm, Sweden; Center for Neuromusculoskeletal Restorative Medicine, Hong Kong Special Administrative Region, China

In recent years, rising life expectancy has led to a significant increase in the prevalence of neurodegenerative disorders, including Alzheimer’s disease (AD), Parkinson’s disease, and age-related cognitive decline. Additionally, other neurological conditions such as glioblastoma, the most common and aggressive brain tumor in adults have been more frequently reported in aging populations. The brain itself is highly vulnerable to age-related changes, particularly disruptions in homeostatic regulation, which further contribute to its functional decline and heightened susceptibility to disease. This has led to a surge of interest in understanding the cellular and molecular mechanisms driving these changes, with a heightened focus directed at microglia, the resident immune sentinels of the brain. Microglia under physiological conditions actively survey the brain environment, clear invading pathogens, remove dead or dying neurons, and promote synaptic remodeling and neuroprotection. They support neuronal functions and overall brain activity in close cooperation with other glial cells, particularly astrocytes, involved in synaptic signal transmission (Malvaso et al., 2023). However, microglia exhibit significant heterogeneity between species and even between brain regions that must be considered when interpreting research findings.

As the brain ages, microglia experience substantial morphological and functional alterations. They shift towards a pro-inflammatory state, increasing the release of cytokines and generating oxidative stress while losing their homeostatic functions. The aging brain creates an environment conducive to this transition by accumulating harmful substances such as lipofuscin, tau, and amyloid-β aggregates, which can act as chronic inflammatory triggers. Additionally, pathology-related mitochondrial dysfunction and oxidative stress can impair microglial function, either by directly affecting cellular metabolism or through persistent activation in response to inflammatory signals. This process establishes a feedback loop where prolonged microglial activation sustains neuroinflammation, eventually leading to a primed state. Over time, these primed microglia lose their ability to appropriate response to a new stimulus, resulting in functional exhaustion and an inability to regulate neural homeostasis effectively (Niraula et al., 2017; Malvaso et al., 2023;). Such sustained microglial activation leads to a loss of their protective functions, thereby contributing to neuronal damage, disrupting neural communication, and accelerating chronic neuroinflammation. This persistent neuroinflammatory state exacerbates the progression of neurodegenerative diseases, including AD and Parkinson’s disease.

Recent advancements in microglial research, particularly by the development of methods on a single-cell resolution, have improved our understanding of the diverse states and phenotypes of microglia occurring under different conditions and disease states. However, age-associated microglial changes remain incompletely understood. Further research is required to determine whether microglia primarily act as responders to immunological insults that occur during brain aging or whether their age-related alterations actively drive disease progression. Additionally, experimental variability between mouse and human models presents significant challenges in translating findings into human neurobiology. Differences in microglial function, gene expression, and immune responses between species must be carefully addressed to ensure that preclinical models accurately reflect human pathology (Olah et al., 2018; Malvaso et al., 2023).

In the following sections, we highlight key differences between findings in human and animal models, examine variations in studies utilizing primary microglial cultures versus acutely isolated microglia, and discuss emerging approaches that may help bridge the gap between model systems and humans.

**Model-dependent variability in microglial senescence induction:** Current literature describing microglia in aging presents often contradictory findings, largely due to variability in experimental models, genetic backgrounds of animal studies, and differences in isolation and culture protocols for primary microglia. These differences significantly affect the observed microglial phenotype, leading to inconsistencies in how age-related changes are characterized. A critical distinction must be made between the culture of primary microglia *ex vivo* and those acutely isolated from the brain or analyzed in brain tissue directly. Primary microglia cultured for an extended period is characterized by the development of a senescent phenotype positive for cell cycle arrest markers (*Cdkn2a* encoding p16, *Cdkn1a* encoding p21, and *Tp53* encoding p53), increased expression of lysosomal senescent markers such as with senescence associated beta-galactosidase, or increased production of pro-inflammatory cytokines (interleukin-1β, interleukin-10, tumor necrosis factor-α, transforming growth factor-β). However, microglia analyzed immediately after isolation or in brain tissue directly show no changes in cell cycle regulators resulting in cell cycle arrest, and maintain lysosomal parameters, with only a mild increase in cytokine release. A similar discrepancy is observed when primary microglia in culture exhibit changes in telomere length over time while microglia in aged brains shows relatively stable telomere length (Stojiljkovic et al., 2019). Another alternative approach of the premature aging model incorporates microglial triple depletion in mice by PLX5622, a pharmacological inhibitor of colony stimulating factor receptor 1, where microglia repopulate the brain following the end of the treatment. This method exploits the fact that microglia can undergo replicative exhaustion. While this model shows similarities to classical aging, notably a downregulation of homeostatic genes and non-senescent phenotype of microglia (Li et al., 2023), further investigation is needed to confirm whether the repopulated cells are truly microglia or other myeloid cells infiltrating the brain following depletion. In humans, only a limited number of studies have investigated differences in the microglial phenotype between younger and older individuals. In particular, human aging studies have yet to fully confirm senescence induction in microglia. One study reported the presence of p16-positive microglia in the brains of aged individuals, although other key senescence markers were notably absent. Furthermore, analysis of aging mouse brains revealed two distinct clusters of p16-positive microglia. The first cluster was age-associated and characterized by the differential expression of genes related to cell cycle regulation and motility. In contrast, the second cluster was found predominantly in younger animals and was associated with the expression of inflammatory genes (Olah et al., 2018; Talma et al., 2021). However, a microglial senescence phenotype, characterized by increased senescence associated beta-galactosidase activity, telomere shortening, and enhanced expression of senescence-related genes such as *Cdkn2a* (encoding p16), *Cdkn1a* (encoding p21), *Il1b* (encoding interleukin-1β), and *Serpine1* (encoding PAI-1), has been observed in microglia located near amyloid-β plaques in APP/PS1 mice, a mouse model of amyloid-β accumulation (Hu et al., 2021).

Another approach utilizes long-term cultivation of the BV-2 microglial cell line (RRID: CVCL_0182), an immortalized line derived from 1-week-old mouse pups through v-raf/v-myc transformation (Škandík et al., 2025), to induce replicative senescence through progressive telomere exhaustion. This model, unlike stress-induced senescence developed after irradiation or chemotherapeutic drug treatment that cause DNA breaks and represent a more damage-like response, reflects naturally occurring changes driven by the gradual exhaustion of cellular systems over time. Although immortalized cell lines have certain limitations, such as induction of proliferation, growing evidence supports the idea that senescence can still be induced in such cells. Recent advancements in cancer research suggest that inducing a senescent state in tumors with deregulated proliferation may serve as a promising therapeutic strategy, with drugs targeting specific senescence markers showing potential for clinical application. The long-term cultured BV-2 microglia do not exhibit classical signs of senescence, such as cell cycle arrest, alterations in the lysosomal-mitochondrial axis, or upregulation of senescence-associated genes, as well as other primary or human microglia (Škandík et al., 2025). This suggests that long-term *in vitro* aged BV-2 cells more closely resemble microglia from aged brains without signs of disease, as they do not express classical senescence markers typically observed in *ex vivo* cultured primary microglia or AD pathology. Supporting this, previous studies have demonstrated that BV-2 microglia transcriptomic profiles and functional implications (nitric oxide production and astrocyte activation) are more similar to hippocampal microglia *in vivo* compared to *ex vivo*-cultured primary microglia (Henn et al., 2009).

**Figure 1:**
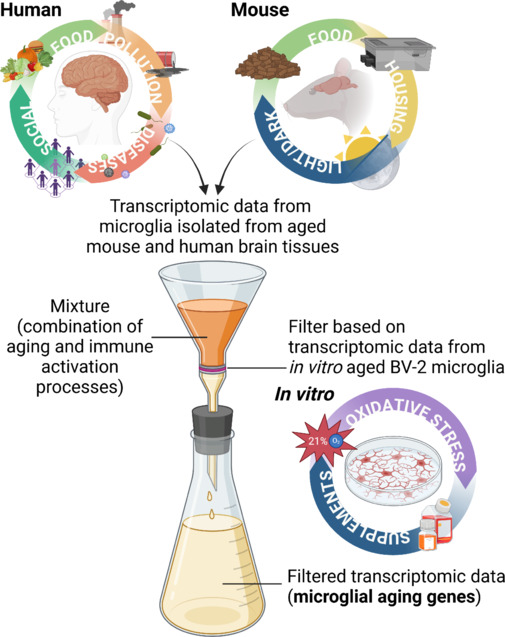
Multi-model approach to evaluate microglial aging. The illustration depicts the impact of various factors on microglial aging across human, animal, and *in vitro* models. The human brain represents the most complex system, influenced by diverse external factors, including dietary habits, environmental pollutants, diseases, and social interactions, all of which contribute to microglial priming over a lifetime. Laboratory mice experience a more controlled environment; however, factors such as diet composition, housing conditions, and light/dark cycles still affect the aging process and microglial state resulting in discrepancies in data from aging studies. Lastly, *in vitro* models, such as long-term cultivated BV-2 microglia, lack the complexity of *in vivo* systems but, due to highly controlled experimental conditions, offer a more specific view of intrinsic cellular changes with minimal extrinsic influence. However, limitations of cell cultures, such as increased oxidative stress due to 21% oxygen exposure and variability in supplement batches, must be considered. Therefore, only a multi-model approach can effectively identify the key drivers of microglial aging, encompassing both intrinsic cellular aging parameters and the broader immune response changes observed in humans and experimental animals. Created with BioRender.com.

**How the environment shapes aging in humans and mouse models:** Human studies, despite valuable insight into microglial aging phenotype, are often confounded by environmental influences such as exposure to pollutants, diet, previous illnesses, accompanying co-morbidities, and affected by sociological factors during the lifetime, which complicate the interpretation of results (**Figure 1**). All these factors, in both mouse and human aging studies, influence microglia throughout their lifespan, resulting in so called primed phenotype, a state where their immune responses become altered compared to earlier reactions (Niraula et al., 2017). As a result, studies do not fully explore microglial aging *per se*, but instead capture microglial immune adaptations and responses accumulated over time. Mutual comparison of mouse aging studies, utilizing C57BL/6 mice from datasets containing 887, 813, and 6701 significant deregulated genes of similar age (23, 24, and 22 months old), show an overlap of 140 genes across all three studies (Škandík et al., 2025). This relatively low overlap can be explained on different housing conditions (**Figure 1**) between studies that affect experimental animals, especially in long-term housing that is accompanied by aging studies (Mieske et al., 2023). In this case, *in vitro* aged BV-2 microglia are cultivated in a more controlled environment with fewer variables, providing valuable insights into microglial cellular aging and minimalizing extrinsic factors that can affect microglial activation. One drawback of BV-2 microglia is their weaker immune response to stimuli compared to brain-resident microglia or primary microglia cultured *in vitro*, despite showing regulation of many of the same genes (Henn et al., 2009). However, aged BV-2 cells exhibit reduced immune activation compared to their younger counterparts when stimulated with bacterial lipopolysaccharide, suggesting a decline in immune competence over time (Škandík et al., 2025). This observation is in agreement with previous studies describing reduced microglial immune response with more advanced age (Olah et al., 2018; Li et al., 2023).

**Using a multiparameter approach to identify true microglial aging drivers beyond immune activation:** A comparative analysis further reveals that lipopolysaccharide-treated BV-2 microglia share nearly three times more overlapping genes with mouse and human studies compared to unstimulated BV-2 microglia. This supports the earlier assertion that many microglial aging studies primarily reflect immunological activation rather than true molecular drivers of aging. Additionally, utilizing *in vitro* engineered aged BV-2 microglia as a reference filter (**Figure 1**) enables the identification of genuine microglial aging markers, distinguishing them from complex immune activation processes acquired over a lifetime when compared with available mouse and human transcriptomic datasets (Škandík et al., 2025).

We propose that future research on microglial aging should adopt a comprehensive, multifaceted approach that integrates *in vitro*, *in vivo*, and human studies. It is crucial to clearly distinguish between microglial aging and immune activation, as current studies often reflect an immunologically primed state rather than true age-associated changes. Long-term culture models, such as BV-2 microglia, offer valuable insights into the molecular processes underlying cellular aging without external stress-induced artifacts. Comparative analyses with mouse and human datasets further emphasize the importance of distinguishing true aging drivers from activation-related changes. By combining data from multiple models and validating findings across platforms, we can overcome existing limitations and variability in aging research. This integrated strategy will not only help unravel the true mechanisms of microglial aging but also lay the foundation for developing more targeted therapeutic interventions. Recent unsatisfactory outcomes of previous therapeutic strategies targeting age-related neurological diseases, aiming at the primary causes of neurodegenerative disorders, such as anti-amyloid therapies for AD and CAR-T therapy for glioblastoma, have failed to demonstrate significant efficacy. These setbacks highlight the need for a paradigm shift in treatment strategies for microglia as a potential therapeutic intervention. Advanced multiparametric research aimed at identifying key drivers of microglial dysfunction will enable the development of precise therapeutic strategies focusing on reprogramming microglial responses to maintain a protective role.

*This work was supported by the Swedish Research Council and the Swedish Brain Foundation, the Cancer Research Funds of Radiumhemmet, the Strategic Research Area in Cancer (StratCan), the Strategic Research Area in Neuroscience (StratNeuro), the Swedish Cancer Society, the Swedish Childhood Cancer Foundation, the Karolinska Institutet Foundation, the InnoHK initiative of the Innovation and Technology Commission of the Hong Kong Special Administrative Region Government (to BJ). Open access funding is provided by the Karolinska Institute.*

**Additional file:** *Open peer review report 1.*

OPEN PEER REVIEW REPORT 1
