# Supplementary material for: Decoding microglial aging through multi-model approaches
Source: Neural Regen Res. 2025 Jun 19;21(6):2315–6. doi: 10.4103/NRR.NRR-D-25-00229 (PMC13211777; doi:10.4103/NRR.NRR-D-25-00229)
Supplement: Supplementary file 1 [file NRR-21-2315_Suppl1.pdf]

OPEN PEER REVIEW REPORT 1

Name of journal: Neural Regeneration Research
Manuscript NO: NRR-D-25-00229
Title: Decoding microglial aging through multi-model approaches
Reviewer's Name: Tino Emanuele Poloni
Reviewer's country: Italy

COMMENTS TO AUTHORS

The topic is very interesting because it could represent a new approach to the pathogenesis of age-related neurodegenerative diseases; especially considering the controversial and overall disappointing results of the "disease-modifying" treatments that are currently available (for example anti-amyloid therapy for AD). I suggest discussing these aspects in the concluding remarks.

I have another suggestion. In the first page of the main text, at lines 33 - 37, the statements are not so clear. The sentence: "Despite growing research, many questions remain unanswered about the exact role of microglia in brain aging... losing their homeostatic functions over time." should be reworded. To make this introductory part more complete, I suggest to point out the following issues: 1) human and mice microglia are highly heterogeneous and there is a significant difference between mice and humans; 2) microglia can protect or damage neurons, depending upon the microenvironment and complex interactions with other glial cells (mainly astrocytes); 3) the loss of homeostatic microglial function may be due either to a loss of function caused by aging-related mitochondrial dysfunction and oxidative stress, or to an excess of activation with production of toxic inflammatory mediators, especially from "primed" cells. In this regard, I suggest quoting the following manuscript: Cells 2023, 12, 2824. https://doi.org/10.3390/cells12242824
